# Clinical performance of a point-of-care assay for measurement of presepsin in patients with bacteremia

**DOI:** 10.1186/cc12958

**Published:** 2013-11-05

**Authors:** Yasuo Fukui, Yoshikazu Okamura

**Affiliations:** 1 Department of Gastroenterological Surgery, Kochi Health Sciences Center, Kochi, Japan; 2 Research and Development Division, Yachiyo R&D Department, Mitsubishi Chemical Medience Corporation, Tokyo, Japan

## Background

The soluble CD14 subtype (sCD14-ST: renamed presepsin), which is approximately 13 kDa, has been identified as a protein whose levels increase specifically in the blood of sepsis patients. In this study, we evaluated the clinical performance of a point-of-care assay for measurement of presepsin in admitted sepsis patients.

## Materials and methods

We obtained 43 cases with blood culture test-positive from patients admitted to our hospital and compared presepsin levels with procalcitonin (PCT), CRP and white blood cell count.

## Results

Positive ratios of presepsin levels of patients with Gram-negative bacterial infection, Gram-positive bacterial infection and fungal infection were higher than those of PCT. When 43 cases were divided into four groups (sepsis, severe sepsis, septic shock and infection groups), presepsin levels were only significantly different between sepsis/infection group and severe sepsis group (*P *< 0.05). Presepsin levels reflected the blood culture test and sepsis severity more than other biomarkers.

## Conclusion

This assay has sufficient clinical performance in patients admitted to the hospital in addition to the emergency room and ICU.

